# Remimazolam for Pediatric Procedural Sedation: Results of an Institutional Pilot Program

**DOI:** 10.3390/jcm12185937

**Published:** 2023-09-13

**Authors:** Tatsuya Hirano, Yoshitaka Kimoto, Norifumi Kuratani, David Cavanaugh, Keira P. Mason

**Affiliations:** 1 Department of Anesthesia, National Hospital Organization Saitama Hospital, Wako 351-0102, Japan; 2 Department of Anesthesiology, Kurume University School of Medicine, Kurume 830-0011, Japan; kimoto_yoshitaka@kurume-u.ac.jp; 3 Department of Anesthesia, Saitama Children’s Medical Center, Saitama 330-8777, Japan; nori-kuratani@umin.ac.jp; 4 Boston Biostatistical Consulting, North Reading, MA 01864, USA; dmcav6@gmail.com; 5 Department of Anesthesia, Critical Care and Pain Medicine, Boston Children’s Hospital, Boston, MA 02115, USA; keira.mason@yahoo.com

**Keywords:** pediatrics, sedation, remimazolam

## Abstract

Remimazolam, an ultra-short-acting benzodiazepine sedative, was first approved in 2020 in Japan as a general anesthetic for adults. However, its utilization in pediatric settings remains unexplored and, to date, is confined to isolated case reports due to a lack of specific pediatric labeling. The primary objective of our study was to evaluate the safety profile of remimazolam when used for procedural sedation in children following dosages established in adult protocols. Additional parameters, including dosage per kg of body weight, duration of the procedure, efficacy (measured as successful completion of the procedure), the necessity for supplemental medications, and changes in physiological parameters, such as the heart rate (HR) and mean arterial blood pressure (MAP), were assessed. Our study encompassed 48 children with an average age of 7.0 years. The objective Tracking and Reporting Outcomes of Procedural Sedation tool indicated no adverse events. In our cohort, propofol and ketamine were used as adjunctive treatments in 8 and 39 patients, respectively, with successful completion of all procedures. Notable hemodynamic variability was observed, with 88.4% of patients experiencing a ≥20% change (increase or decrease) and 62.8% experiencing a ≥30% change in MAP. Additionally, a ≥20% change in HR was observed in 54.3% of patients, and a ≥30% change was observed in 34.8% of patients. Nevertheless, none of the patients required pharmacological intervention to manage these hemodynamic fluctuations. Our findings suggest that remimazolam, when supplemented with propofol or ketamine, could offer a safe and effective pathway for administering procedural sedation in pediatric populations.

## 1. Introduction

Remimazolam, an ultra-short-acting benzodiazepine sedative, has emerged as an innovative anesthetic option worldwide. It was approved for use as a general anesthetic in Japan, China, Korea, and the USA and for additional applications, including monitored anesthesia care and sedation, in several European Union countries. This drug represents the first Federal Drug Administration (FDA)-approved sedative since the launch of dexmedetomidine over two decades ago. In adult populations, remimazolam demonstrates numerous advantages, such as an impressive half-life of approximately 0.92 h [1], fewer adverse events, faster recovery, and quicker return to cognitive function when compared with propofol and midazolam [1,2,3].

While these advancements have expanded anesthesia options for adults, the utility of remimazolam in pediatric populations remains underexplored and poses certain challenges. A nationwide pediatric trial under the FDA’s supervision started in April 2021; however, obtaining pediatric-specific labeling is a process that may take several years (ClinicalTrials.gov Identifier: NCT04851717; https://clinicaltrials.gov/ct2/show/NCT04851717 (accessed on 30 March 2023)). Currently, remimazolam is approved exclusively for adult use, and its off-label application in the pediatric context is restricted to isolated case reports [4,5].

Given the limited scope of remimazolam’s use in pediatrics, there is an urgent need for detailed examination and dissemination of pediatric experiences with this drug. While our previous work touched upon remimazolam’s application in general anesthesia in a pediatric cohort [6], specific investigations into its efficacy and safety in pediatric sedation are sparse. This study aims to bridge this knowledge gap by presenting the outcomes of the first-ever pediatric sedation program using remimazolam, with dosages extrapolated based on approved adult labeling. This initiative will contribute significantly to the current literature and could potentially aid in the expansion of remimazolam’s approved indications to include pediatric sedation.

## 2. Materials and Methods

This study was approved by the Institutional Review Committee of Saitama Children’s Medical Center (Approval Code: 2022-05-025). From August 2020 to December 2022, remimazolam was utilized for pediatric procedural sedation at a tertiary-care, university-affiliated pediatric hospital. The institutional Committee on Pharmaceutical Affairs granted approval for off-label pediatric application of remimazolam as part of routine anesthesia and sedation practices. An institutional review board sanctioned the evaluation of electronic medical records from the perioperative period (pre-, intra-, and postoperative) as a component of a continuous quality assurance initiative, waiving the necessity for informed consent.

We included all patients under 18 years who underwent procedures with remimazolam sedation at our institution between August 2020 and December 2022. Patients with airway devices, such as tracheal intubation or supraglottic airway devices, were excluded. The primary study outcome was to establish the incidence and variety of adverse events as specified using the Tracking and Reporting Outcomes of Procedural Sedation tool (TROOPS). This internationally developed instrument facilitates objective identification of the severity of adverse events (minor, intermediate, and sentinel) and provides standardized benchmarks for delineating sedation outcomes [7,8]. Secondary outcomes included dosage, efficacy (measured as the ability to successfully complete the procedure), administration of supplemental medications, and physiological variations in heart rate (HR) and mean arterial blood pressure (MAP).

While there was no fixed protocol for remimazolam dosing, anesthesiologists determined the dosage based on the adult recommendations provided in the remimazolam package insert from Japan, administering it using continuous intravenous (IV) infusion at a rate of 12 mg/kg/h until achieving the desired Ramsay sedation score of 3. After induction, the sedation depth was maintained in the range of 1–2 mg/kg/h at the anesthesiologist’s discretion. The use of ketamine, fentanyl, and propofol was left to the anesthesiologist’s discretion to reach or maintain the targeted sedation depth. Remimazolam boluses of 0.2 mg/kg were administered intermittently as needed to sustain a Ramsay sedation score of 3. Information on flumazenil doses and additional sedative medications was gathered from electronic medical records.

### Statistical Analysis

Given that this was a retrospective study, a rigid sample size calculation was not necessitated. Nevertheless, as our primary outcome was safety, an estimate of remimazolam sedation safety based on our sample size was imperative. Utilizing the conventional rule of 3, if no adverse events are observed in 48 patients, the upper boundary of the 95% confidence interval for the incidence of adverse events can be calculated as 3/48 = 0.06 = 6%. Hence, if the true incidence of adverse reactions due to remimazolam sedation exceeded 6%, we would expect to observe at least one adverse reaction in our sample of 48 cases.

As a retrospective case series analysis, our study used descriptive statistics for data scrutiny. We extracted information from patients’ medical records, summarizing it using appropriate statistical methods, contingent on the data type. Key demographic information summarized included the number of patients, age, weight, body mass index (BMI), and the American Society of Anesthesiologists (ASA) score, along with baseline HR and MAP. Procedure-related details, such as the duration of the procedure, length of remimazolam infusion, and the time from the last dose of remimazolam to discharge, were also gathered and summarized. Changes in MAP and HR, specifically incidences of 20% and 30% variations from the baseline (either increase or decrease), were accounted for and summarized using frequency counts. Additionally, we recorded the number of patients who required supplemental medications, including propofol, ketamine, and fentanyl, and reported the respective doses administered. We also examined the number of instances where flumazenil was administered to facilitate emergence. All statistical analyses were conducted using SAS^®^ (version 9.4, SAS^®^ Institute, Cary, NC, USA).

## 3. Results

We recorded data for 48 children with a median (range) age of 7.0 (0.1,17.8) years. The median (range) American Society of Anesthesiologists score was 2.0 (1.0,3.0), with five patients scoring 1, thirty-five scoring 2, and eight scoring 3. Table 1 illustrates demographic and baseline characteristics, including any supplemental medications administered.

In the majority of cases, remimazolam alone was insufficient for sedation. A notable 95% of patients received remimazolam in combination with other sedatives or analgesics, such as ketamine, propofol, or fentanyl. Propofol and ketamine, either alone or in combination, were given at the anesthesiologist’s discretion. Specifically, propofol was given as a bolus at the start of sedation and between procedures. Ketamine was given as a continuous IV infusion in 22 cases and as a bolus in 17. Overall, 3 cases used propofol alone, 34 used ketamine alone, and 5 used both.

We recorded no adverse events categorized as “Sentinel” in the TROOPS. Although milrinone and atropine were administered to one patient each, these were unrelated to circulatory changes resulting from remimazolam. Specifically, the patient given milrinone had constrictive cardiomyopathy, and milrinone was used irrespective of the sedation procedure. Atropine administration did not correlate with any particular bradycardia, and the rationale for its use remained indeterminable from electronic records. We inferred that the anesthesiologist likely incorporated it as a part of routine induction. Thus, the use of these drugs does not classify under “Vasoactive Drug Administration” in the TROOPS “Sentinel” category.

While other anesthetics were often needed in conjunction with remimazolam for effective sedation, this study primarily aimed at evaluating the safety of remimazolam use rather than its standalone efficacy. Hence, the need for other sedatives is not classified as “Sedation Insufficient of Intermediate” in TROOPS. Some patients were given flumazenil for early awakening post-procedure, not for emergency airway complications, so its use does not fall under “Intermediate” in TROOPS either. Consequently, none of the patients were categorized as “Intermediate” in TROOPS.

Regarding mean arterial blood pressure (MAP), we managed to collect data from 43 out of 48 patients. We calculated the percent change from baseline MAP for each patient’s lowest and highest recorded MAP. The number of patients in each group was measured by dividing them into three groups: those with little change from baseline (0% to 20% change), those with moderate change from baseline (20% to 30% change), and those with large change from baseline (30% or greater).

**Figure 1:**
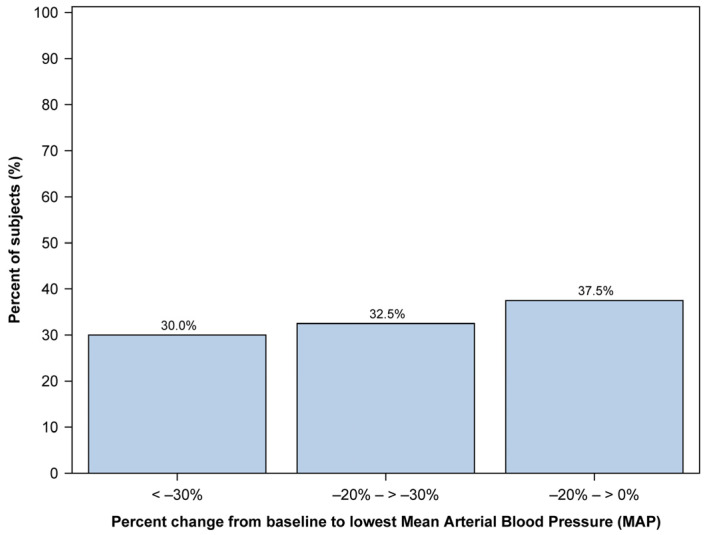
Distribution of the lowest mean arterial pressure (MAP) change from baseline observed in the 40 pediatric patients during the procedure. The *x*-axis represents the percentage change in MAP, while the *y*-axis represents the percentage of subjects. MAP change is divided into three distinct groups: 0% to −20% change, −20% to −30% change, and −30% or greater change.

The median percent change from baseline to the lowest MAP was −22.6% (range: −58.2% to 19.2%). Among these, 40 patients (93.0%) had a drop in MAP below their baseline. The distribution of percent changes from baseline to the lowest MAP is illustrated in Figure 1. The distribution of the percent change in the lowest MAP values was almost even.

**Figure 2:**
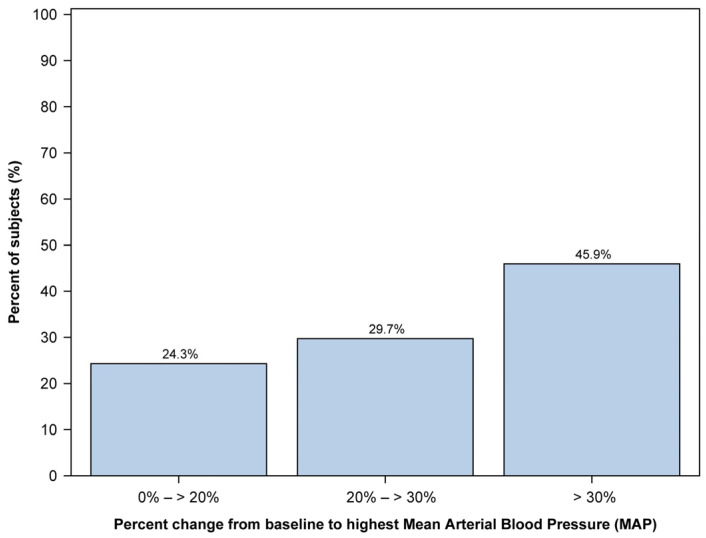
Distribution of the highest mean arterial pressure (MAP) change from baseline observed in the 37 pediatric patients during the procedure. The *x*-axis represents the percentage change in MAP, while the *y*-axis represents the percentage of subjects. MAP change is divided into three distinct groups: 0% to 20% change, 20% to 30% change, and 30% or greater change.

The median (range) percent change from baseline to the highest MAP was 26.4 (−21.9, 67.3). There were 37 (86.0%) patients with an increase in the highest MAP and 6 (14.0%) without an increase. The distribution of percent changes from baseline to the highest MAP is illustrated in Figure 2. The distribution of the highest MAP values increased in a stepwise manner from the group with little change to the group with large change.

A significant finding was that 38 (88.4%) patients experienced a ≥20% change (increase or decrease) and 27 (62.8%) patients experienced a ≥30% change in MAP (lowest or highest) from baseline.

Regarding heart rate (HR), we managed to collect data from 46 out of 48 patients. We calculated the percent change from baseline HR for each patient’s lowest and highest recorded HR. The number of patients in each group was measured by dividing them into three groups: those with little change from baseline (0% to 20% change), those with moderate change (20% to 30% change), and those with large change (30% or greater).

**Figure 3:**
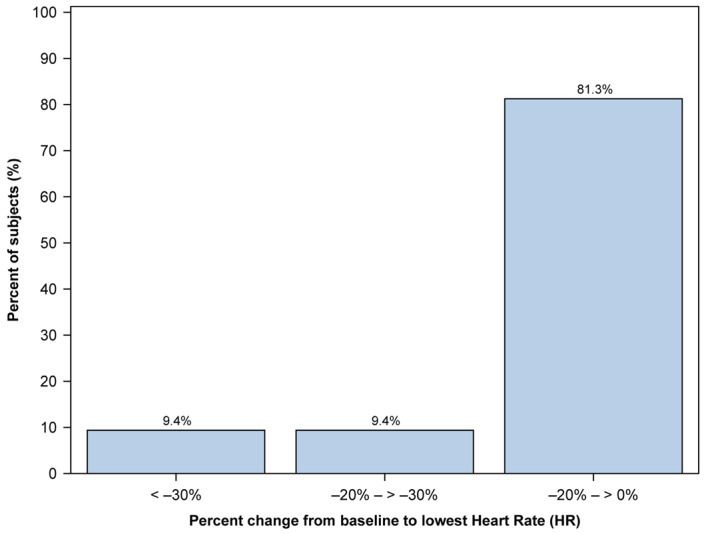
Distribution of the lowest heart rate (HR) changes from baseline observed in 32 pediatric patients during the procedure. The *x*-axis represents the percentage change in HR, while the *y*-axis represents the percentage of subjects. HR change is divided into three distinct groups: 0% to −20% change, −20% to −30% change, and −30% or greater change.

The median (range) percent change from baseline to lowest HR was 6.7 (−71.4, 53.3). Of the 46 patients, 32 (69.6%) experienced a decrease in HR below their baseline, whereas 14 (30.4%) showed no decrease. The distribution of percent changes from baseline to the lowest HR is illustrated in Figure 3. Most of the percent changes in the lowest HR values were distributed among the groups with little change from baseline.

**Figure 4:**
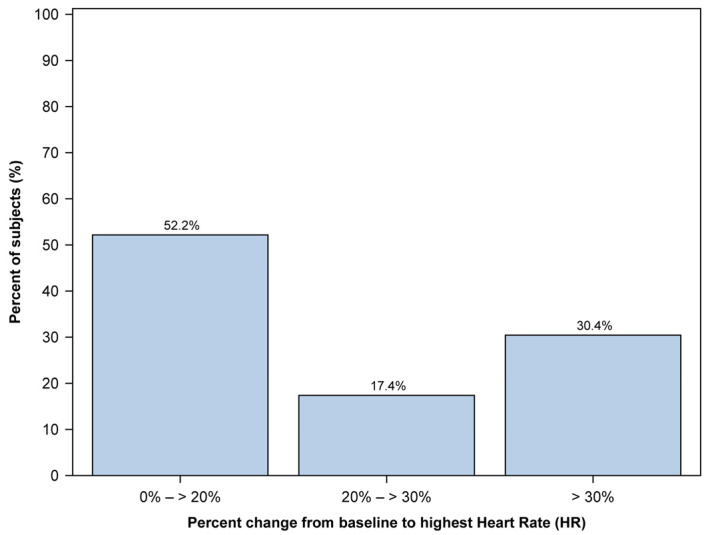
Distribution of the highest heart rate (HR) change from baseline observed in 46 pediatric patients during the procedure. The *x*-axis represents the percentage change in HR, while the *y*-axis represents the percentage of subjects. HR change is divided into three distinct groups: 0% to 20% change, 20% to 30% change, and 30% or greater change.

The median (range) percentage difference from the baseline to the highest HR was recorded as 18.4 (2.3, 113.3). All 46 patients whose data could be collected showed an increase in their highest HR. The distribution of percent changes from baseline to the highest HR is illustrated in Figure 4. Half of the percent changes in the lowest HR values were distributed among the groups with little change from baseline. On the other hand, there were a few groups with large changes.

There were 25 (54.3%) patients who experienced a ≥20% change (increase or decrease) and 16 (34.8%) who had a ≥30% change in HR from baseline (lowest or highest).

Notably, none of the patients required pharmacologic intervention for hemodynamic fluctuations. Moreover, out of the 15 patients who had a MAP and HR ≥ 20%, 13 (86.7%) had received ketamine. Furthermore, among the 22 patients with an increase HR ≥ 20%, 19 (86.3%) had received ketamine. Among the 28 patients with an increase MAP ≥ 20%, 26 (92.9%) also had received ketamine.

The mean (standard deviation: SD) and median (interquartile range: IQR) duration of the procedure was 57.8 (43.62) minutes and 54.5 (35.5–73) minutes, respectively. The mean (SD) and median (IQR) duration of remimazolam administration were 85.5 (53.3) minutes and 84.5 (44.25–113.75) minutes, respectively. The mean (SD) and median (IQR) remimazolam dosage administered were 0.08 (0.190) and 0.03 (0.24–0.42) mg/kg/min, respectively. Remimazolam administered was calculated by dividing the total dose of remimazolam administered during sedation by the procedure time (min) and patient weight (kg). Table 2 displays the results concerning the procedure and remimazolam usage.

## 4. Discussion

In our pilot program, remimazolam was primarily used for radiological imaging studies such as magnetic resonance imaging and angiography, which are typically accompanied by minimal noxious stimulation. In adults, remimazolam has demonstrated a faster onset, predictable influence on the electroencephalogram and delta waves, and a more rapid return to baseline neurocognitive function when compared with midazolam [9]. Differences in pharmacokinetic and pharmacodynamic responses across nationalities have been observed, highlighting the importance of international collaborations [10]. Future research should explore the wider use of remimazolam for procedures such as bronchoscopy [11], gastrointestinal endoscopies [12], and oral surgery [13] in the pediatric population. Larger studies are necessary to ascertain the broad clinical utility and limitations of remimazolam in the pediatric population, as well as its effects on post-procedure recovery and return to functionality.

In our study, a significant majority of our patients, precisely 95%, were administered remimazolam alongside other sedatives or analgesics such as ketamine, propofol, or fentanyl. This common co-administration could indicate that remimazolam, while effective, might not provide sufficient sedation or analgesia when used as a single agent for certain pediatric procedures. However, the choice of adjunct sedatives or analgesics should be carefully considered as they could potentially influence the hemodynamic parameters and recovery profile of the patients. This co-administration also raises interesting questions about the potential interactive effects of remimazolam with other sedatives or analgesics, and how these combinations could influence the recovery process. For instance, a subset of our patients received flumazenil at the end of the procedure to hasten their return to cognitive function. This approach has proven effective in speeding up psychomotor recovery and restoring baseline hemodynamics in adults who received midazolam or remimazolam [14]. Therefore, it would be valuable to determine if the observed hemodynamic and psychomotor responses to flumazenil in the pediatric population receiving remimazolam along with other sedatives or analgesics are comparable to those documented in adults.

In our findings, an 18.4% median increase in the highest HR was reported, which was lower than the 28% change reported in adult remimazolam trials [9]. Nearly all our patients who showed an increase in HR and MAP were administered ketamine. The observed increases could be attributed to the sympathomimetic effect of ketamine, which is often used to enhance sedation conditions and counteract the bradycardic and hypotensive effects of other sedatives [15,16,17]. Variability in HR and blood pressure in response to other intravenous (IV) anesthetics and sedatives such as propofol and dexmedetomidine is common, typically requires no pharmacological intervention, and is of little clinical consequence [18,19,20,21,22,23]. Sedation studies in adults comparing hemodynamic responses between remimazolam and propofol suggest that only remimazolam preserves the balance between sympathetic and parasympathetic activity during induction [24]. Future pediatric studies should aim to determine the incidence and degree of remimazolam-induced hypotension and the potential for mitigating these effects with the use of ketamine or other anesthetics.

The Tracking and Reporting Outcomes of Procedural Sedation tool has been used in both adult and pediatric sedation studies to provide a standardized and objective way to describe and classify the occurrence and severity of adverse events [8]. In our study population, no adverse events were recorded. We did observe fluctuations in hemodynamic (MAP and HR) values in almost all patients, with an increase in MAP and a decrease in HR being the most common. Our findings of 22.6% median decrease in the lowest MAP with remimazolam are similar to those reported in adult trials, which show an average decrease of 24% in the lowest MAP [9]. A meta-analysis of adults sedated with remimazolam for colonoscopy revealed a 36% incidence of hypotension [25].

The worldwide introduction and regulatory approval of remimazolam have varied, with inconsistencies in dosing, administration methods (bolus versus infusion), and indications (induction and maintenance of general anesthesia versus procedural sedation). In the United States, European Union, and China, remimazolam is approved for adult procedural sedation. The most frequently approved adult dose of remimazolam for procedural sedation is 5 mg via IV injection over 1 min, with a rescue dose of 2.5 mg via IV injection over 15 s [11]. In contrast, in Japan and South Korea, remimazolam is approved for general anesthesia (not for sedation), at doses that differ from those approved for sedation [26,27,28]. The dose of remimazolam recommended for their anesthesia induction is an infusion rate of up to 12 mg/kg/h, with a maintenance infusion of 1–2 mg/kg/h [29].

With its approval by regulatory authorities occurring less than three years ago, remimazolam is still a relatively novel substance in terms of clinical use, experience, and the scope of clinical trials (whether published, ongoing, or planned). As with other sedatives and anesthetics, the approved adult dosing may need adjustments for pediatric applications. For instance, in 1999, dexmedetomidine was approved for adult sedation at a bolus IV dosage of 1 mcg/kg, with continuous infusions up to 0.7 mcg/kg/h to maintain procedural sedation. Over the past 20 years, this dosing regimen has been increased when administered in off-label doses for pediatric procedural sedation [30,31,32,33,34,35,36].

This study has several limitations. As a retrospective study, potential inconsistencies and discrepancies in data acquisition and reporting may exist. The investigators carefully reviewed the electronic medical records for any aberrant data and found no significant inconsistencies. Until there is approved pediatric labeling for remimazolam, prospective trials will be limited or challenging. The scientific community will need to launch innovative pilot programs like ours for data collection and outcome analysis. We used TROOPS to evaluate the safety of remimazolam. However, the safety evaluation was not based on the use of remimazolam alone, but on the combination of remimazolam and other drugs. A study using remimazolam alone may be needed to truly evaluate safety. Hemodynamic variability was compared to each patient’s baseline vital signs. In the pre-procedure period, the baseline MAP and HR may not accurately represent a patient’s standard vital signs due to anxiety. As a result, the baseline MAP and HR may be falsely elevated in some (or all) patients, thereby skewing the data calculated from these baselines. An alternative would be to measure these patients’ hemodynamics during natural sleep, but this was not possible in our study as the patients underwent day procedures. The hemodynamic variability observed in our study could partly be a pharmacodynamic response to ketamine. Since remimazolam alone does not provide any analgesic or anesthetic properties, adjunct sedatives may be necessary for painful procedures. Evaluations will be needed for these adjunct medications, not only for their efficacy but also for their safety and recovery profile.

## 5. Conclusions

In our pilot studies at the institution, remimazolam in combination with other sedatives or analgesics presented promising potential as an effective, efficient, and safe IV sedative for pediatric procedural sedation. While sedation with remimazolam was often performed in combination with other medications, it enabled procedures lasting up to 60 min. Multicenter international trials are necessary to assess the full range of clinical utility, pharmacokinetic and pharmacodynamic responses, and recovery profile of remimazolam, both as a standalone and in combination with other sedatives, anesthetics, and analgesics.

## Figures and Tables

**Table 1:** Demographic and baseline characteristics of pediatric patients that underwent sedation with remimazolam and a summary of concomitant drug administration.

Variable	Statistics (n = 48)	
Age (yrs)	Median	7.0
	Min, MAX	0.1, 17.8
Weight (kg)	Median	16.7
	Min, max	3.7, 74.2
BMI	Median	16.1
	Min, MAX	6.7, 26.8
ASA score	Median	2
	Min, MAX	1.0, 3.0
ASA score, n (%)	1	5 (10.4)
	2	35(72.9)
	3	8 (16.7)
Baseline heart rate (bpm)	Median	100.0
	Min, MAX	60.0, 132.0
Baseline MAP (mmHg)	Median	64
	Min, MAX	53.0, 84.0
Type of procedure n (%)	Computerized tomography (CT)	3 (6.3)
	Magnetic resonance imaging (MRI)	10 (20.8)
	Radiation therapy	4 (8.3)
	Intravenous angiography	28 (58.3)
	Miscellaneous	3 (6.3)
Propofol (mg) (n = 8)	Mean (SD)	44.4 (71.54)
	Median	17.5
	Min, MAX	10.0, 220.0
Ketamine (mg) (n = 39)	Mean (SD)	95.9 (64.50)
	Median	90
	Min, MAX	1.0, 225.0
Fentanyl (mg) (n = 1)	Mean (SD)	0.03 (NA)
	Median	0.03
	Min, MAX	0.03, 0.03
Flumazenil (mg) (n = 5)	Mean (SD)	0.2 (0.07)
	Median	0.2
	Min, MAX	0.1, 0.3

**Table 2:** Summary of procedure duration, remimazolam administration, and dosage per unit time per body weight.

Variable	Statistics	
Duration of Procedure (min)	Mean (SD)	57.8 (43.62)
	Median (IQR)	54.5 (35.5–73)
Duration of remimazolam (min)	Mean (SD)	85.5 (53.3)
	Median (IQR)	84.5 (44.25–113.75)
Remimazolam administered (mg/kg/min)	Mean (SD)	0.08 (0.190)
	Median (IQR)	0.03 (0.24–0.42)

Standard deviation: SD, interquartile range: IQR.

## Data Availability

Data sharing is not applicable to this article. This was approved by the Institutional Review Board of Saitama Children’s Medical Center.
